# Role of epicardial adipose tissue in human atrial fibrillation

**DOI:** 10.1002/joa3.12825

**Published:** 2023-02-19

**Authors:** Naohiko Takahashi, Ichitaro Abe, Shintaro Kira, Yumi Ishii

**Affiliations:** ^1^ Department of Cardiology and Clinical Examination Oita University Faculty of Medicine Oita Japan

**Keywords:** angiopoietin‐like protein 2, atrial fibrillation, computed tomography images, epicardial adipose tissue, fibrotic remodeling

## Abstract

A recent meta‐analysis among which four reports were conducted in Japan demonstrated that epicardial adipose tissue (EAT) is closely associated with an increased risk of atrial fibrillation (AF) recurrence after catheter ablation. We previously investigated the role of EAT in AF in humans. Left atrial (LA) appendage samples were obtained from AF patients during cardiovascular surgery. Histologically, the severity of fibrotic EAT remodeling was associated with LA myocardial fibrosis. Total collagen in the LA myocardium (i.e., LA myocardial fibrosis) was positively correlated with proinflammatory and profibrotic cytokines/chemokines, including interleukin‐6, monocyte chemoattractant protein‐1, and tumor necrosis factor‐α, in EAT. Human peri‐LA EAT and abdominal subcutaneous adipose tissue (SAT) were obtained by autopsy. EAT‐ or SAT‐derived conditioned medium was applied to the rat LA epicardial surface using an organo‐culture system. EAT‐conditioned medium induced atrial fibrosis in organo‐cultured rat atrium. The profibrotic effect of EAT was greater than that of SAT. The fibrotic area of the organo‐cultured rat atrium treated with EAT from patients with AF was greater than in patients without AF. Treatment with human recombinant angiopoietin‐like protein 2 (Angptl2) induced fibrosis in organo‐cultured rat atrium, which was suppressed by concomitant treatment with anti‐Angptl2 antibody. Finally, we attempted to detect fibrotic EAT remodeling on computed tomography (CT) images, which demonstrated that the percent change in EAT fat attenuation was positively correlated with EAT fibrosis. Based on these findings, we conclude that the percent change in EAT fat attenuation determined using CT non‐invasively detects EAT remodeling.

## Graphical abstract

Fibrotic epicardial adipose tissue remodeling.

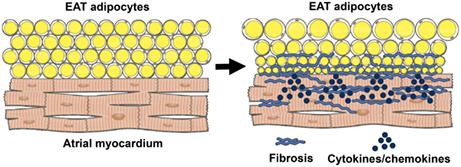

## 1 INTRODUCTION

Atrial fibrillation (AF) is the most common type of sustained arrhythmia in the clinical setting.^1^ Catheter ablation dramatically reduces the burden of AF and its associated symptoms^2^; however, AF recurrence after catheter ablation is still a critical problem.^3^ A strong association between obesity and a high AF recurrence after catheter ablation has been demonstrated previously,^4^ and the role of adiposity in the development and maintenance of AF has attracted much attention. A previous study showed that the variable risk of AF is associated with different adipose tissue compartments.^5^ Among them, the association increases progressively from overall, abdominal, pericardial, and epicardial adiposity, respectively.^5^ Interestingly, Mahajan et al. reported that epicardial fat accumulation and subsequent fatty infiltration are associated with an inhomogeneous reduction in voltage and conduction abnormalities, leading to AF development.^6^ It has been reported that epicardial adipose tissue (EAT) volume, as assessed by computed tomography (CT) or magnetic resonance imaging, is associated with the presence and recurrence of AF.^7^, ^8^ EAT may exacerbate atrial myocardial fibrosis, which is a critical substrate for AF.^9^ In this special review article, we first review the association between EAT and AF recurrence after catheter ablation, before introducing the experimental approaches that we have used to clarify the role of EAT in AF.^10^, ^11^, ^12^

## 2 EAT AND AF RECURRENCE AFTER CATHETER ABLATION

Previous studies have examined the association of EAT with the risk of AF recurrence after catheter ablation. Recently, Chen et al. performed a systematic review and meta‐analysis to examine the association between EAT and AF recurrence after catheter ablation.^13^ Ten studies that included a total of 1840 patients with AF were included, among which four reports were conducted in Japan.^14^, ^15^, ^16^, ^17^ In their 2011 report, Nagashima et al^14^ obtained three‐dimensional volume‐rendered reconstructed images of the EAT (total EAT) and the EAT surrounding the left atrium (peri‐LA EAT) using 320‐row multidetector CT in 40 patients with paroxysmal AF (PAF) or persistent AF (PeAF) (PAF, *n* = 24; PeAF, *n* = 16) who underwent catheter ablation, as well as 37 age‐matched controls. Both the total EAT volume and the peri‐LA EAT volume increased progressively from control to PAF to PeAF, respectively. The EAT volume was independently associated with the presence of AF after adjustment for possible confounding factors. The EAT volume was greater in patients with AF with post‐ablation recurrence than in patients without recurrence.^14^ Moreover, in their 2015 report, Masuda et al^15^ measured the total and peri‐LA EAT volumes by 320‐detector‐row multislice CT. Fifty‐three consecutive patients with drug‐refractory AF who were scheduled to undergo catheter ablation were recruited. During the follow‐up period of 16 ± 4 months, early and late recurrence occurred in 29 (55%) and 12 (23%) patients, respectively. The peri‐LA EAT volume was larger in patients with early recurrence than in those without early recurrence. However, there was no difference in the total EAT volume between the two groups. The multivariate analysis revealed that a large peri‐LA EAT volume, PeAF, and a large LA volume were independent predictors of early recurrence. Interestingly, there was no significant difference in peri‐LA or total EAT volume between patients with and without late recurrence. Masuda et al^15^ concluded that the abundance of peri‐LA EAT independently predicted early recurrence after AF ablation. In their 2018 report, Maeda et al^16^ evaluated the relationship between EAT volume and the characteristics of AF, the impact of EAT volume on recurrent AF after radiofrequency ablation, and the cut‐off point for recurrent AF using the receiver operating characteristic (ROC) curve. In 218 consecutive symptomatic patients who underwent ablation for AF (PAF, *n* = 143; PeAF, *n* = 78), the EAT volume index (EAT volume ÷ body surface area, ml/m^2^) was measured using 320‐row multidetector CT. The high EAT volume group showed specific cardiometabolic derangements, as well as LA dilatation and left ventricular (LV) dysfunction. The multivariate regression analysis showed that the EAT volume was an independent predictor of AF recurrence after catheter ablation. A high EAT volume index of ≥85 ml/m^2^ or an EAT volume index cut‐off ≥116 ml/m^2^ predicted AF recurrence after catheter ablation, independent of other risk factors. Maeda et al. concluded that the EAT volume index was an independent predictor of AF recurrence after catheter ablation.^16^ Moreover, in their 2020 report, Kawasaki et al^17^ investigated the relationship of the combination of cardiac sympathetic nerve activity (CSNA) and EAT with AF recurrence 3 months after catheter ablation. Sixty‐four patients with PAF without heart failure were included. Cardiac metaiodobenzylguanidine (MIBG) scintigraphy was performed at baseline and at 3 months after catheter ablation. The MIBG washout rate (WR) was calculated by MIBG imaging. The total EAT volume and the peri‐LA EAT volume were measured by CT before catheter ablation, and the peri‐LA to total EAT volume ratio (P/T) was obtained. The WR change from baseline to 3 months after catheter ablation (dWR) and the P/T were greater in patients with AF recurrence than in those without AF recurrence. The greater dWR and P/T values determined by the ROC curve analysis were independently associated with AF recurrence. The peri‐LA EAT volume was significantly correlated with the baseline WR. Based on these observations, Kawasaki et al. concluded that the combination of dWR and P/T was associated with AF recurrence in patients without heart failure. They suggested that both CSNA and EAT might be related to AF development. The systematic review^13^ included these four studies from Japan, revealing that EAT volume was associated with a higher risk of AF recurrence after catheter ablation and that EAT‐related thickness was a risk factor for AF recurrence after catheter ablation. A sub‐analysis showed that the EAT was strongly associated with a higher risk of AF recurrence in the Asian population, patients aged ≤60 years, and those followed up for more than 1 year (*p* = .020). Based on these observations, Chen et al^13^ concluded that EAT‐related thickness seems to be the marker most strongly associated with a greater risk of AF recurrence after catheter ablation. They also suggested that EAT‐related thickness should be included in risk stratification to predict AF recurrence before catheter ablation.

The EAT contains abundant ganglionated plexuses (GPs),^18^ which has been implicated in atrial fibrillation recurrence after catheter ablation. Nakahara et al^19^ demonstrated in PeAF patients that pulmonary vein antrum isolation followed by 3‐dimensional reconstructed CT map‐guided EAT‐based ablation efficiently eliminated high dominant‐frequency (DF) sources and yielded relatively high success. Because an overlap between the EAT and high‐DF sites was found, they suggested that an ablation strategy guided by EAT localization may eliminate triggered activity enhanced by cardiac autonomic activation associated with inflammatory EAT.^19^

## 3 OBSERVATIONS IN HUMAN LA APPENDAGE (LAA)

EAT fibrosis (or fibrotic EAT remodeling) is a phenotype that is strongly associated with atrial myocardial fibrosis and AF. Haemers et al^20^ obtained right atrial (RA) samples to show that AF is associated with fibrotic EAT remodeling. However, the main site that demonstrated development and maintenance of AF was the LA.^21^ We therefore attempted to clarify whether fibrotic EAT remodeling occurs in the LA and is associated with atrial myocardial fibrosis.^10^ We also attempted to identify the inflammatory cytokines/chemokines that predominantly promote fibrotic EAT remodeling.^10^

### 3.1 Fibrotic EAT remodeling

**FIGURE 1:**
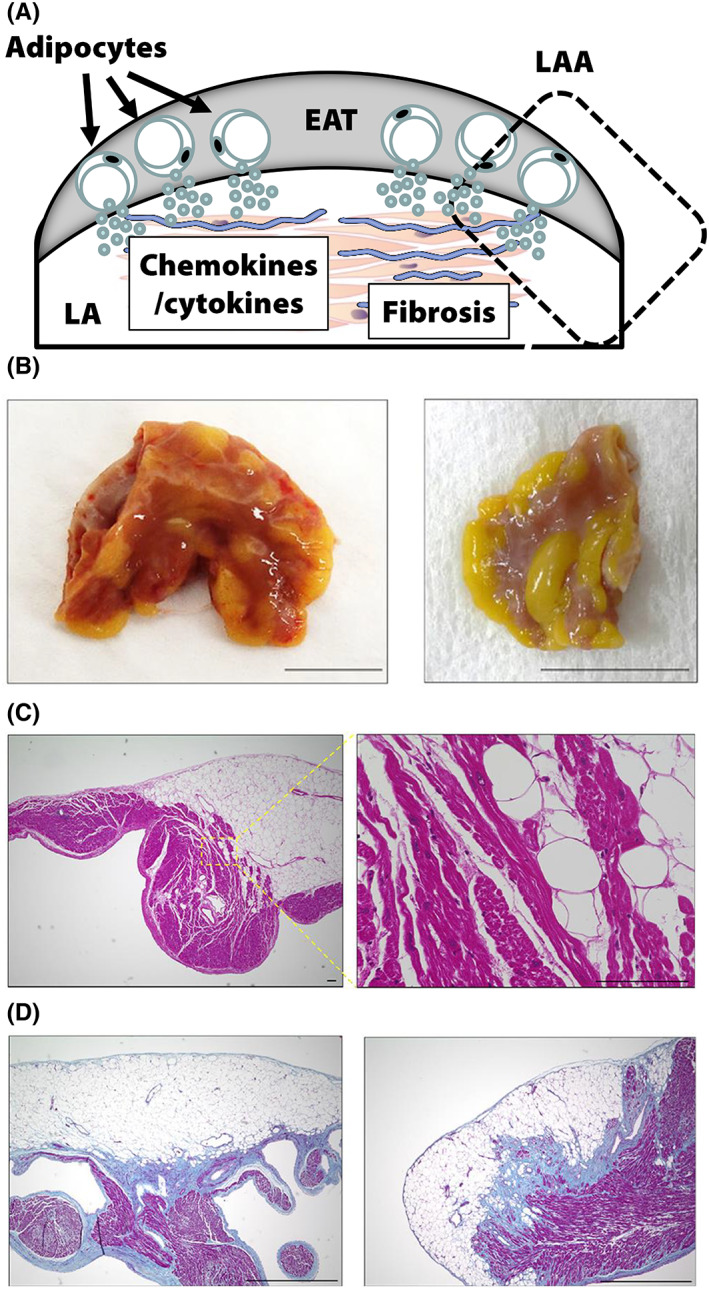
EAT infiltration toward the atrial myocardium. (A) Concept of our study. (B) Macroscopic findings of the excised LAA. The EAT was commonly observed in human LAA samples. Scale bar = 1 cm. (C) Infiltration of the subepicardial area reached the endocardium in some cases. Scale bar = 100 μm. (D) Massive interstitial fibrosis was observed in the atrial myocardium at the junction with the EAT. Scale bar = 1000 μm. EAT, epicardial adipose tissue; LAA, left atrial appendage. (Figure adapted with permission^10^).

**FIGURE 2:**
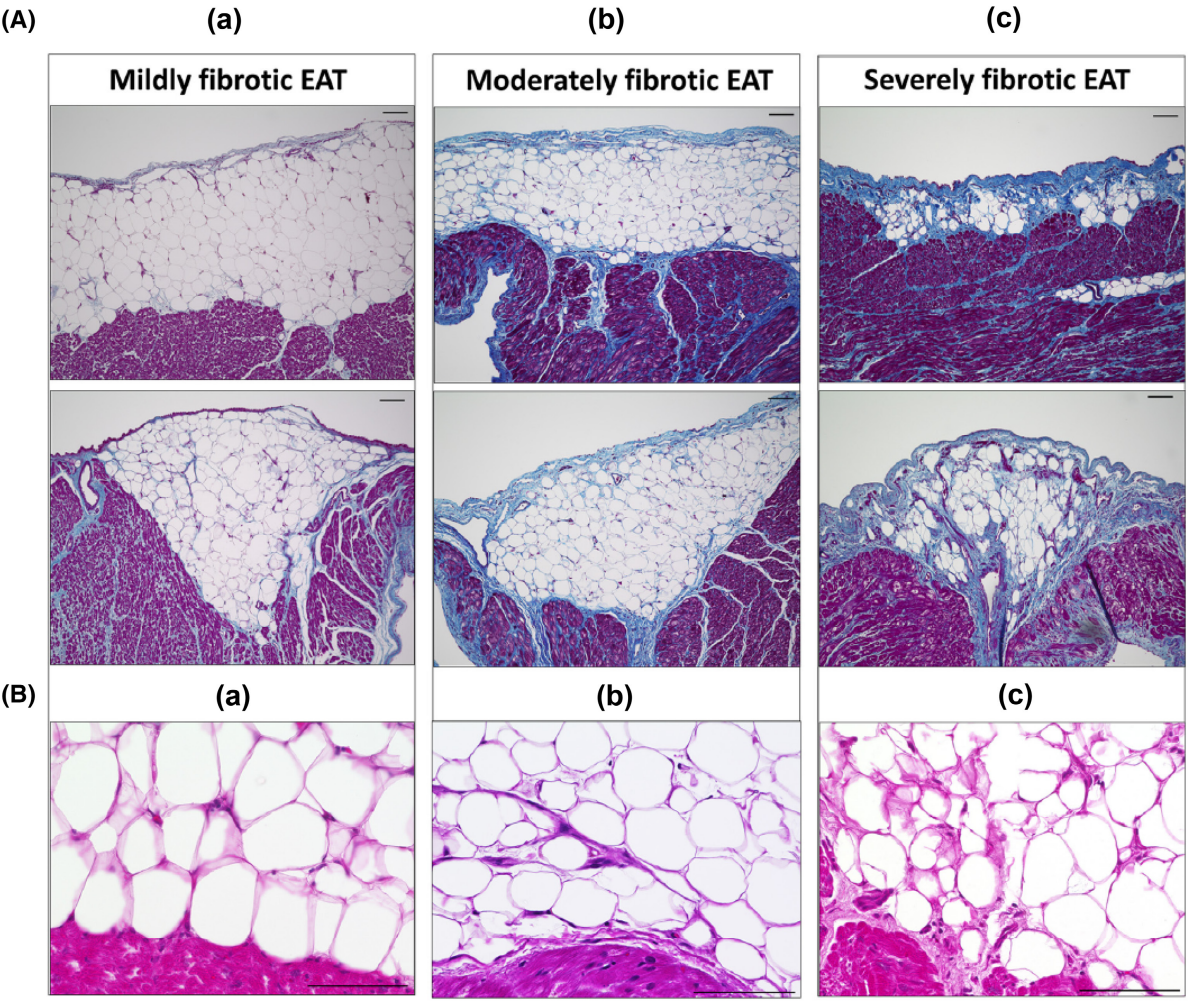
Fibrotic EAT remodeling and its relationship with atrial myocardial fibrosis. (A) Representative Masson's trichrome‐stained images of mildly (a), moderately (b), and severely (c) fibrotic EAT. (B) Hematoxylin and eosin‐stained images of the corresponding EAT. Scale bar = 100 μm. (Figure adapted with permission^10^).

**FIGURE 3:**
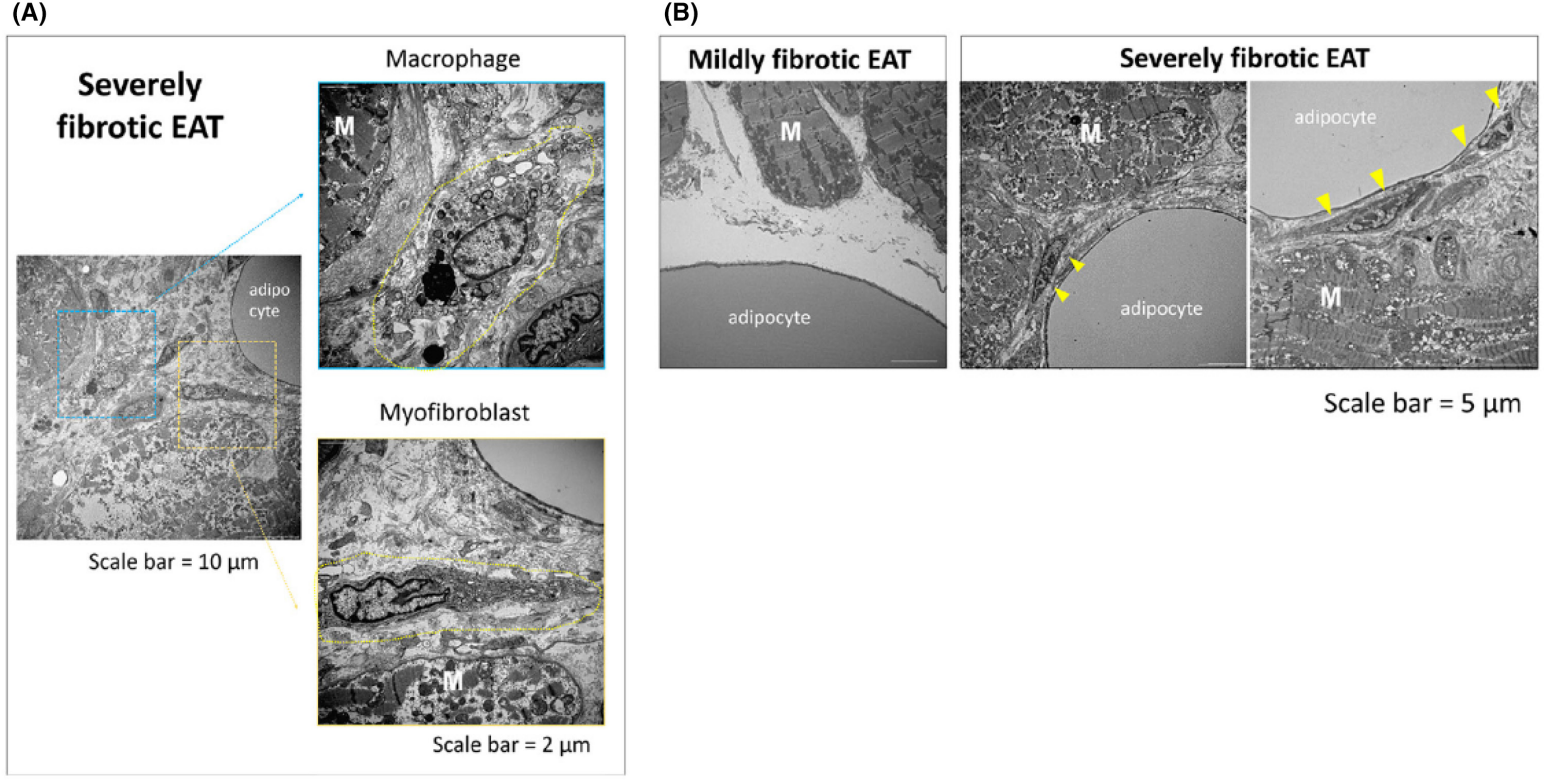
Comparative transmission electron microscopy findings between severely fibrotic EAT and mildly fibrotic EAT. (A) Macrophage and myofibroblast infiltration was observed around adipocytes in severely fibrotic EAT. (B) Stromal cells and myofibrillar disarray were observed at the junction with the adipocytes in severely fibrotic EAT. EAT, epicardial adipose tissue; M, myocardium. (Figure adapted with permission^10^).

Figure 1A depicts the concept of our study.^10^, ^11^, ^12^ The EAT contains abundant adipocytes that secrete various types of proinflammatory cytokine/chemokine. Using human LAA samples, we examined the relationship between the EAT profile and atrial myocardial fibrosis by histological and biochemical analyses in 59 consecutive patients.^10^ Figure 1B shows the representative macroscopic findings of excised LAA in two patients. Each excised LAA sample was surrounded by EAT to some extent. Microscopic findings of LAA tissue sections stained with hematoxylin and eosin revealed that the EAT infiltrated into the atrial myocardium (Figure 1C). Masson's trichrome staining revealed that atrial myocardial fibrosis was predominantly observed at the junction between the EAT and the atrial myocardium (Figure 1D). During the analysis, we noticed remarkable individual variations in EAT fibrotic remodeling. Each EAT could be classified according to the characteristic fibrosis (Figure 2). We divided 59 LAA sections into tertiles according to the severity of EAT fibrosis (mild, moderate, and severe). As shown in Figure 2A(a), mildly fibrotic EAT was characterized by few collagen fibers. Figure 2A(c) shows that severely fibrotic EAT was characterized by abundant collagen fiber accumulation (Figure 2A(c)). Moderately fibrotic EAT demonstrated intermediate features between those of mildly fibrotic and severely fibrotic EAT (Figure 2A(b)). Adipocytes in mildly fibrotic EAT were of similar size, regularly formed, and associated with few stromal cells (Figure 2B(a)), whereas adipocytes in moderately fibrotic EAT were slightly irregular with few stromal cells (Figure 2B(c)). The stromal cells were identified as macrophages and myofibroblasts. Figure 3 demonstrates the results of transmission electron microscopy. In a patient with severely fibrotic EAT, macrophages and myofibroblasts were present between adipocytes and the myocardium (Figure 3A). As shown on the left side of Figure 3B, in a patient with mildly fibrotic EAT, there were few collagen fibers between adipocytes and the myocardium. The myocardial ultrastructure, including the mitochondria, was well preserved. However, in a patient with severely fibrotic EAT, stromal cell infiltration and myofibrillar disarray were observed at the interface between adipocytes and the atrial myocardium (right side of Figure 3B). The myocardial ultrastructure, including the mitochondria, was destroyed.

Interestingly, it has been reported that macrophage infiltration was enhanced in the peri‐LV EAT of patients with coronary artery disease (CAD), and that infiltration was positively correlated with CAD severity.^22^ Taken together, it was suggested that macrophage infiltration was involved in fibrotic remodeling of the peri‐LA EAT, and atrial myocardial fibrosis^10^ was very similar to coronary artery atherosclerosis promoted by peri‐LV EAT.^22^

### 3.2 Proinflammatory cytokines/chemokines in the EAT

We evaluated the protein content of pro‐inflammatory and pro‐fibrotic cytokines/chemokines in the EAT, as well as the protein content of collagen in the atrial myocardium.^10^ The protein content of total collagen in the atrial myocardium was positively correlated with the protein content of interleukin (IL)‐1β, IL‐2, IL‐6, IL‐7, IL‐9, IL‐12, eotaxin, granulocyte‐macrophage colony‐stimulating factor, monocyte chemoattractant protein (MCP)‐1, macrophage inflammatory protein‐1β, RANTES, and tumor necrosis factor (TNF)‐α, respectively. In contrast, the protein content of total collagen in the atrial myocardium was not correlated with the serum cytokine/chemokine concentration. These results indicate that local inflammation between the EAT and the atrial myocardium could underpin atrial myocardial fibrosis to a greater degree than systemic inflammation.

With respect to the pathology of EAT fibrosis, it is logical to consider that the composition of the EAT would play an important role. Mazurek et al^23^ examined cytokines/chemokines by comparing the EAT with subcutaneous adipose tissue (SAT). The authors observed that the concentrations of IL‐1, IL‐6, MCP‐1, and TNF‐α in the EAT were greater than in the SAT, suggesting that cytokines/chemokines in the EAT might predominantly contribute to CAD. In our study, the EAT was obtained from the LAA in patients with AF, while in Mazurek et al's^23^ study, the EAT was obtained from the proximal right coronary artery in patients with CAD. It is interesting that the cytokines/chemokines detected in their study wholly included the cytokines/chemokines that we detected in the peri‐LA EAT.^10^ Taken together, these findings suggest that these cytokines/chemokines in peri‐coronary artery EAT and peri‐LA EAT similarly influence the progression of atherosclerosis and atrial myocardial fibrosis.

### 3.3 Role of angiopoietin‐like protein‐2 (Angptl2) and hypoxia‐inducible factor (HIF)‐1α in EAT

**FIGURE 4:**
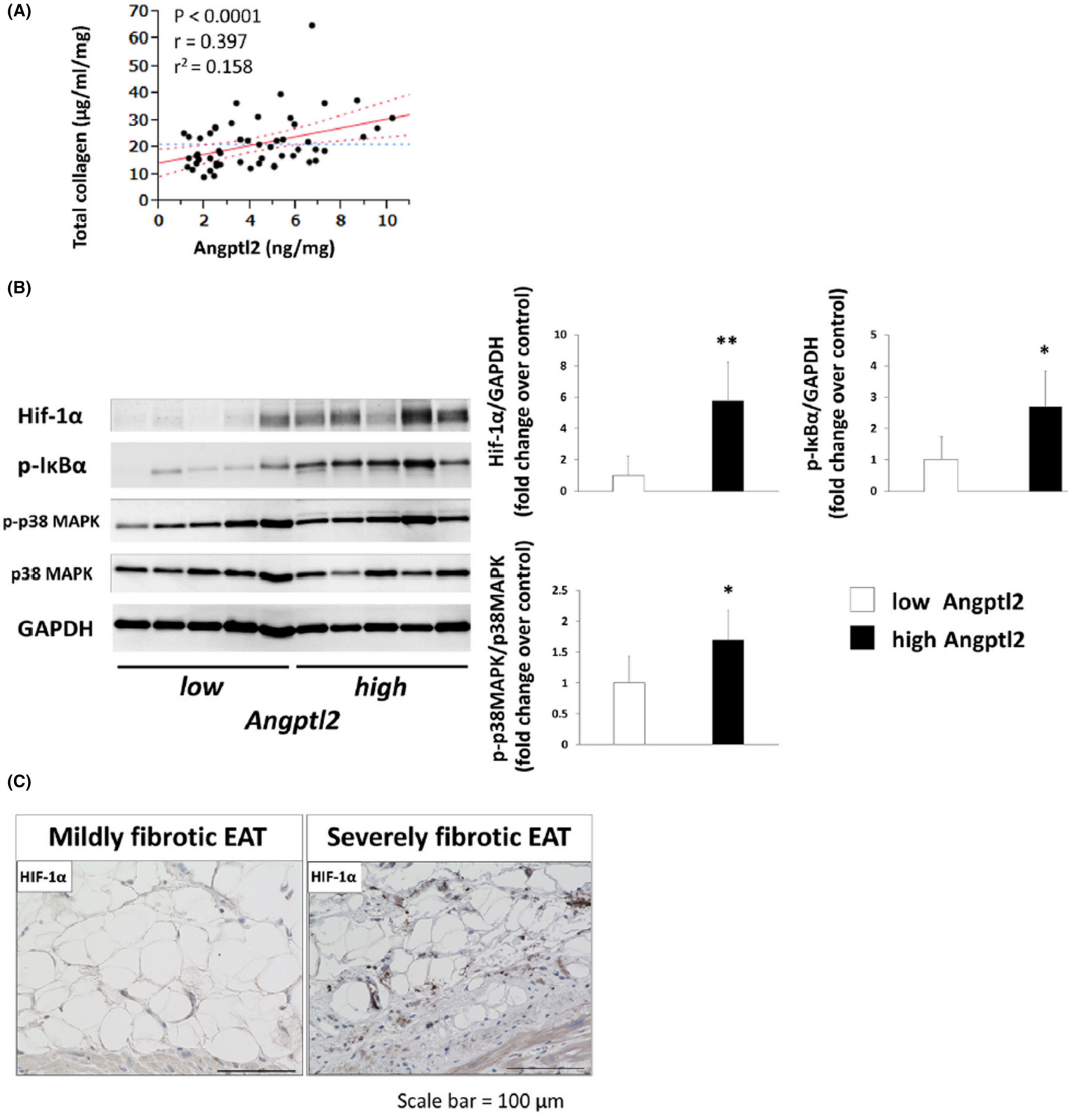
Expression of Angptl2 and HIF‐1α in the EAT. (A) Positive correlation between total collagen in the atrial myocardium and Angptl2 in the EAT. (B) Protein expression of HIF‐1α, phospho‐IκBα, phospho‐p38 MAPK, p38 MAPK, and GAPDH in the EAT of the high and low Angptl2 groups (*n* = 5 per group). The data are presented as the mean ± SD. *p < .05, **p < .01 vs. low Angptl2 group. (C) Representative immunohistochemical staining for HIF‐1α in the mildly and severely fibrotic EAT groups. Angptl2, angiopoietin‐like protein‐2; EAT, epicardial adipose tissue; HIF‐1α, hypoxia‐inducible factor‐1α; p‐p38 MAPK, phospho‐p38 mitogen‐activated protein kinase; GAPDH, glyceraldehyde‐3‐phosphate dehydrogenase. (Figure adapted with permission^10^).

As shown in Figure 4A, total collagen in the atrial myocardium was positively correlated with Angptl2 in the EAT. When patients were divided into two groups according to the protein concentration of Angptl2 in the EAT, the protein expression of HIF‐1α, phospho‐IkBα, and phospho‐p38 mitogen‐activated protein kinase (MAPK)/p38 MAPK, which are known upstream and downstream factors of Angptl2 in the EAT, was greater in patients with high Angptl2 expression than in those with low Angptl2 expression (Figure 4B). In addition, areas that were positive for HIF‐1α were abundant in the severely fibrotic EAT but were scarcely observed in the mildly fibrotic EAT (Figure 4C).

Angptl2 is a member of the Angptl family.^24^ It is secreted primarily by adipose tissue, and its expression is regulated by hypoxia.^25^ Angptl2 overexpression induces chronic inflammation and adipose tissue remodeling.^26^ Furthermore, Angptl2 may contribute to the development of abdominal aortic aneurysm by exacerbating proinflammatory signaling.^27^ Downstream of Angptl2, an integrin α_5_β_1_/p38 MAPK pathway underpinning the expression of matrix metalloproteinases (MMPs) and promoting IkB degradation and nuclear localization of nuclear factor‐kB for the expression of inflammation‐related genes was reported previously.^26^ Based on these findings, we investigated whether Angptl2 secreted from the EAT might be involved in the qualitative alteration of EAT and myocardial fibrosis. One report examined Angptl2 in human EAT,^28^ showing that Angptl2 expression in the peri‐LV EAT was correlated with TNF‐α expression in patients with CAD.^28^ Only one study has investigated Angptl2 in patients with AF,^29^ showing that the mRNA expression of Angptl2 in the peri‐RA EAT tended to increase, but the increase did not reach statistical significance in patients with AF compared with patients in sinus rhythm.^29^ In terms of Angptl2 expression, the role of HIF‐1α should be discussed. HIF‐1α in adipose tissue induces a transcriptional program that enhances the synthesis of extracellular matrix components, leading to adipose tissue fibrosis.^30^ It has been reported that rapid expansion of adipocytes causes local hypoxia, which induces HIF‐1α expression. In addition, a previous report has shown that cytokines/chemokines and Angptl2 are upregulated by hypoxia.^31^ Despite the lack of evidence, HIF‐1α as an upregulator of Angptl2 may be involved in the inflammatory and fibrotic alteration of EAT, which could be important in the context of cardiovascular disease.^32^ Our study demonstrated that fibrotic remodeling and cytokines/chemokines in the peri‐LA EAT were associated with atrial myocardial fibrosis as a substrate for AF.^10^ Our results also suggested that HIF‐1α and Angptl2 overexpression may be involved in these processes.

## 4 ORGANO‐CULTURE SYSTEM TO INDUCE ATRIAL FIBROSIS BY HUMAN EAT

**FIGURE 5:**
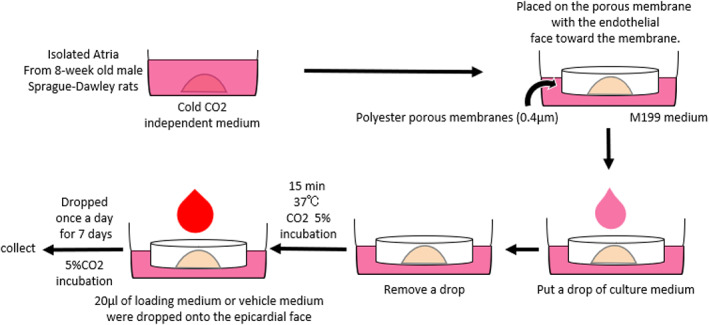
Organo‐culture system. See text for details. (Figure adapted with permission^11^).

We demonstrated that the protein expression of Angptl2 in the EAT was positively correlated with the protein expression of proinflammatory and profibrotic cytokines/chemokines in the EAT and the total collagen content in the atrial myocardium.^10^ Therefore, we used the organo‐culture system to test the hypothesis that Angptl2 in the peri‐LA EAT induces atrial myocardial fibrosis.^11^ The methods are briefly shown in Figure 5. The hearts of 8‐week‐old male Sprague–Dawley rats were rapidly excised under anesthesia. Isolated LA tissue samples were transferred into cold carbon dioxide (CO_2_)‐free medium. Inserts containing polyester porous membranes (pore size, 0.4 μm; Millicell, Merck Millipore) were filled with 2 ml M199 culture medium (Gibco, Invitrogen) containing insulin, transferrin, and sodium selenite (ITS, 1/1000, Sigma‐Aldrich), 5% fetal bovine serum, 10‐mM glucose, 1‐nM isoproterenol, and 100 U/ml penicillin/streptomycin. The isolated atria were placed on the porous membranes with the endothelial side facing toward the membrane. A drop of culture medium was applied to the atria and incubated for 15 min at 37°C (5% CO_2_). After pre‐incubation, 20 μl loading medium (the ratio of conditioned medium to M199 culture medium was 1:4) or vehicle medium (consisting entirely of M199 culture medium) was dropped onto the epicardial side of the atrium once per day and incubated for 7 days (37°C, 5% CO_2_).

### 4.1 Histological analysis

**FIGURE 6:**
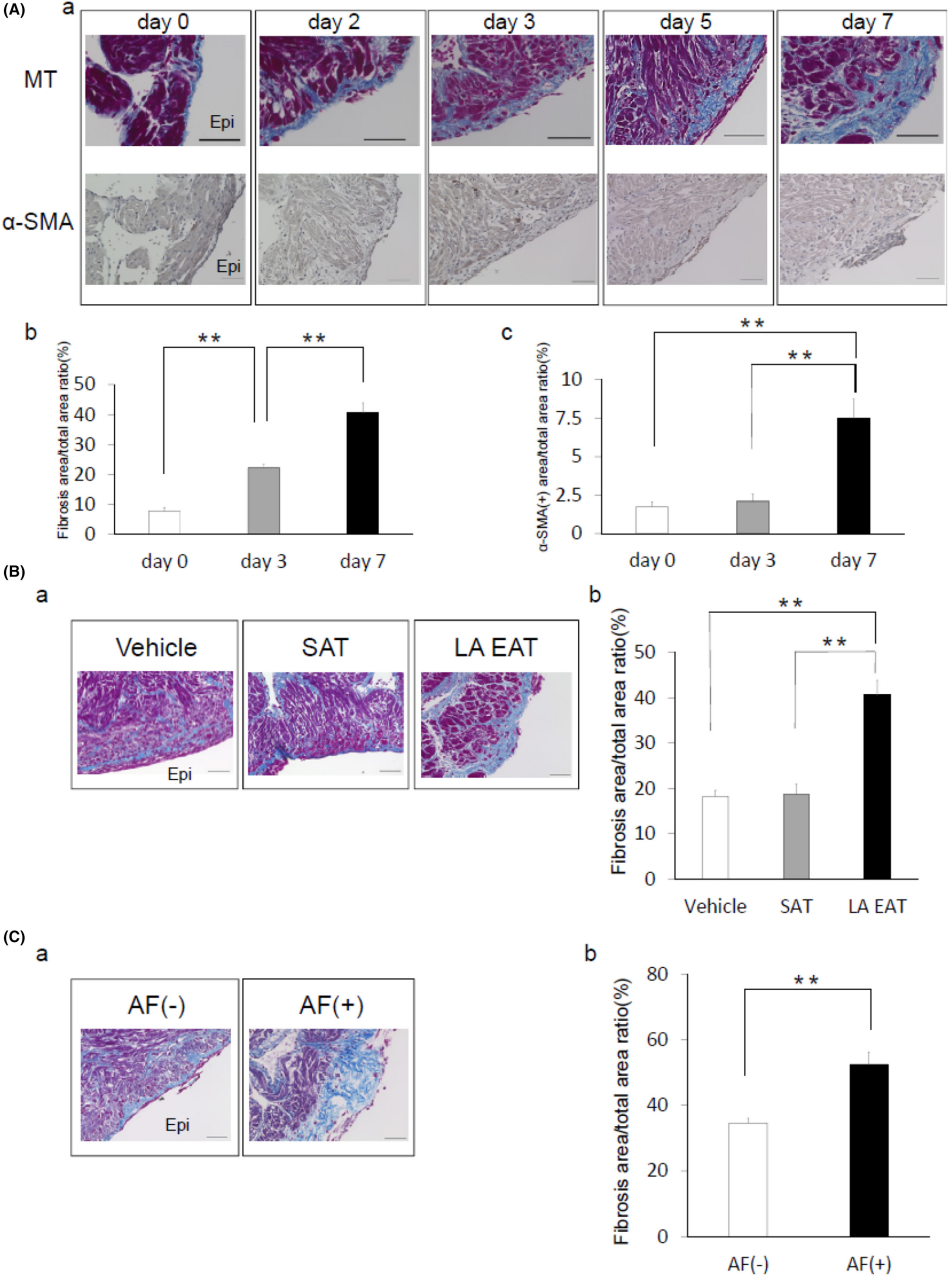
Effects of human EAT and SAT on organo‐cultured rat atrial fibrosis. (A) (a) Representative results of Masson's trichrome and immunohistochemical staining using α‐SMA antibody. Scale bar = 550 mm. (b) Quantification of epicardial fibrosis. The data are presented as the mean ± SEM (*n* = 6 for day 0; *n* = 9 for days 3 and 7). **p < .01. (c) Quantification of α‐SMA‐positive cells (*n* = 6 for day 0; *n* = 9 for days 3 and 7). **p < .01. (B) (a) Representative results of Masson's trichrome staining treated with vehicle‐, SAT‐, or EAT‐conditioned medium for 7 days. Scale bar = 50 μm. (b) Quantification of epicardial fibrosis (*n* = 6 for the vehicle group; *n* = 9 for the SAT and EAT groups). (C) (a) Representative results of Masson's trichrome staining treated with the EAT obtained from patients with or without AF for 7 days. Scale bar = 50 μm. (b) Quantification of epicardial fibrosis (*n* = 6 for the AF (−) group; *n* = 3 for the AF (+) group). **p < .01. α‐SMA, α‐smooth muscle actin; AF, atrial fibrillation; Epi, epicardium; peri‐LA EAT, peri‐left atrial epicardial adipose tissue; MT, Masson's trichrome; SAT, subcutaneous adipose tissue. (Figure adapted with permission^11^).

**FIGURE 7:**
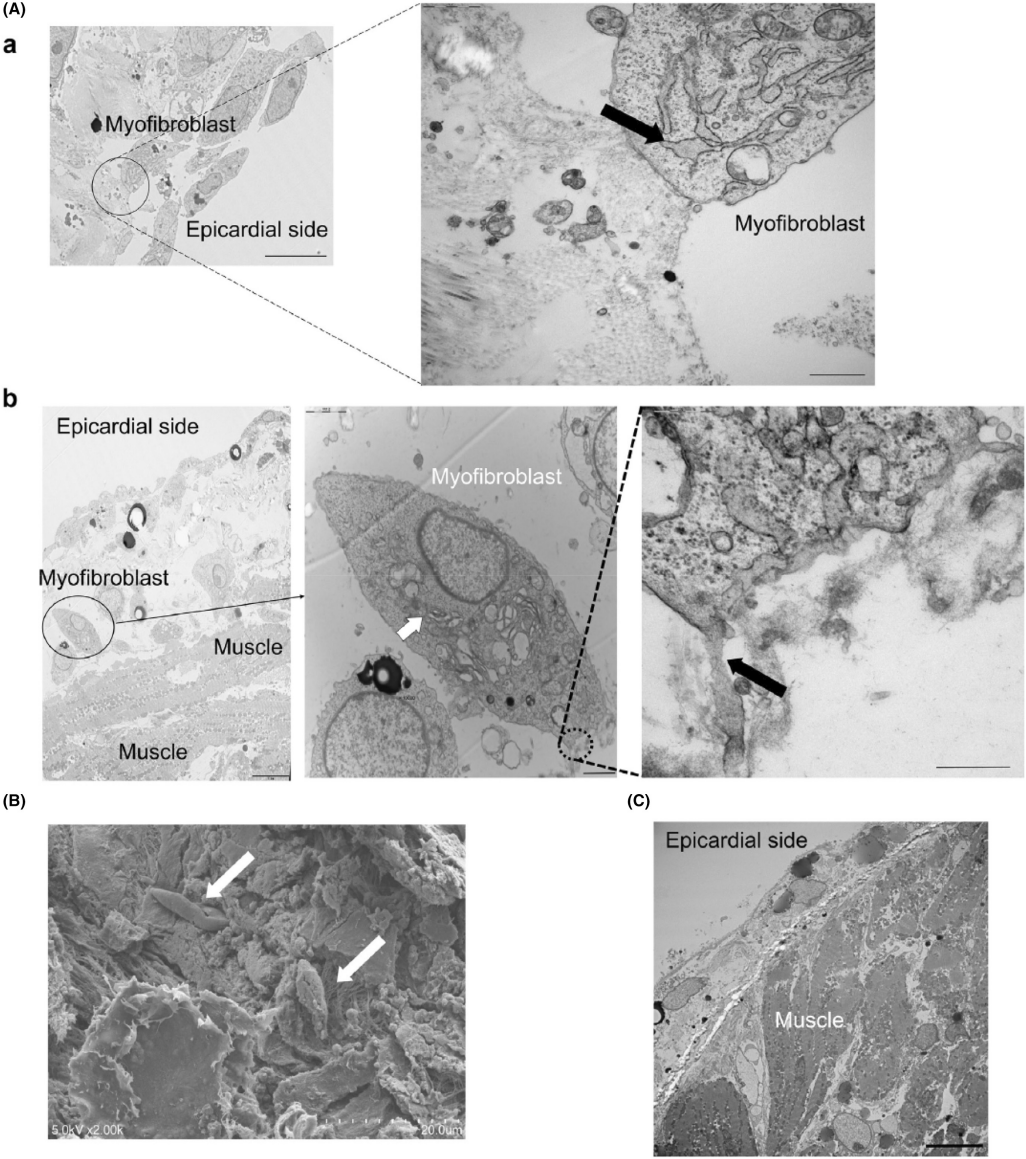
Transmission electron microscopy findings. (A) Transmission electron microscopy findings of organo‐cultured atria treated with the EAT‐conditioned medium for 7 days. A cell with a well‐developed granular endoplasmic reticulum was observed (black arrow (a)). A cell with Golgi body (white arrow) and actin fibers produced from this cell (black arrow) was observed (b). Scale bar for (a) and left panel in (b) = 10 μm. Scale bars for center and right panels = 2 μm and 500 nm, respectively. (B) Scanning electron microscopy images of organo‐cultured atria treated with the EAT‐conditioned medium for 7 days. Spindle‐shaped cells (white arrows) were specifically observed. (C) Myofibroblasts were rarely seen in the atria treated with the SAT‐conditioned medium. Scale bar = 10 μm. (Figure adapted with permission^11^).

Figure 6A(a) shows representative Masson's trichrome staining and immunohistochemical staining results using α‐smooth muscle actin (SMA) antibody in rat atria treated with EAT‐conditioned medium. Human EAT induced fibrosis in the organo‐cultured atria from the epicardial side (Figure 6A(b)), which was associated with an increase in the number of α‐SMA‐positive cells (Figure 6A(c)). Figure 6B(a) shows representative Masson's trichrome staining of the atria treated with vehicle‐, SAT‐, or EAT‐conditioned media for 7 days. The fibrotic area of the SAT‐treated atrium was comparable to that of the vehicle‐treated atrium. However, the fibrotic area of the EAT‐treated atrium was greater than that of the vehicle‐ and SAT‐treated atria (Figure 6B(b)). Figure 6C(a) shows representative Masson's trichrome staining of the EAT obtained from patients with or without AF for 7 days. As shown in Figure 6C(b), the fibrotic area of the organo‐cultured rat atrium treated with EAT from patients with AF was greater than in patients without AF. Figure 7A shows the transmission electron microscopy findings of organo‐cultured atria treated with EAT‐conditioned medium for 7 days. Figure 7A(a) shows that a specific cell type was observed at the epicardial site. High magnification revealed a well‐developed granular endoplasmic reticulum (right side of Figure 7A(a)), and Golgi body (center of Figure 7A(b)) and actin fibers were produced (right side of Figure 7A(b)). The cell type was identified as a myofibroblast. Figure 7B shows the scanning electron microscopy findings. The white arrows show that morphologically identified myofibroblasts were observed at the epicardial site of the organo‐cultured atria treated with the EAT‐conditioned medium for 7 days. In the time‐dependent evaluation, an increase in the number of epicardial myofibroblasts on transmission electron microscopy images was not observed on days 1 and 3. The number of myofibroblasts began to increase on day 5, which continued up to day 7. Conversely, the SAT‐conditioned medium did not induce the appearance of myofibroblasts (Figure 7C).

In our previous study,^11^ the profibrotic effects of the peri‐LA EAT were evaluated using a unique organo‐culture system. Venteclef et al^33^ obtained EAT and SAT samples from patients undergoing coronary bypass surgery. Using a similar organo‐culture technique, they demonstrated that the EAT rather than the SAT induced global fibrosis in the rat atria. Although our observations are consistent with those findings, Venteclef et al^33^ collected the EAT from the LV groove. Our study demonstrated that the peri‐LA EAT induced atrial fibrosis.^11^ In fact, the EAT from patients with AF more effectively induced atrial fibrosis than did EAT from patients without AF (Figure 6C). Our study also demonstrated that the progression of atrial fibrosis induced by EAT was associated with an increase in the number of α‐SMA‐positive cells (Figure 6A). Together with the results of transmission electron microscopy (Figure 7), these α‐SMA‐positive cells were identified as myofibroblasts. The origin of these myofibroblasts could not be identified; however, myofibroblasts were rarely observed in the organo‐cultured rat atria treated with the SAT‐conditioned medium or vehicle. Thus, one possibility is that resident fibroblasts were transformed into myofibroblasts as a result of proinflammatory stimuli from the EAT‐conditioned medium. The other possibility is that the myofibroblasts originated from the migration and transformation of mesenchymal stem cells stimulated by the EAT^34^ because no blood contamination was present in the organo‐culture system. Transmission electron microscopy captured the moment that actin fibers were produced and released from the myofibroblasts, in which the well‐developed granular endoplasmic reticulum and Golgi body were observed (Figure 7A(b)). These characteristic findings were specifically observed in the organo‐cultured rat atrium treated with the EAT‐conditioned medium. It is possible that the process of active myofibroblast infiltration and resultant production of actin fibers may contribute to the initial step of human LA fibrosis induced by the peri‐LA EAT in a living body. Recently, the concept of atrial cardiomyopathy has been established as any complex of structural, architectural, contractile, or electrophysiological change that affects the atria with the potential to produce clinically relevant manifestations.^35^ Our organo‐culture system can reproduce atrial fibrotic changes in patients with AF, which is a major manifestation of atrial cardiomyopathy, thus contributing to the understanding of the mechanisms underpinning human atrial cardiomyopathy.

### 4.2 Role of Angptl2

**FIGURE 8:**
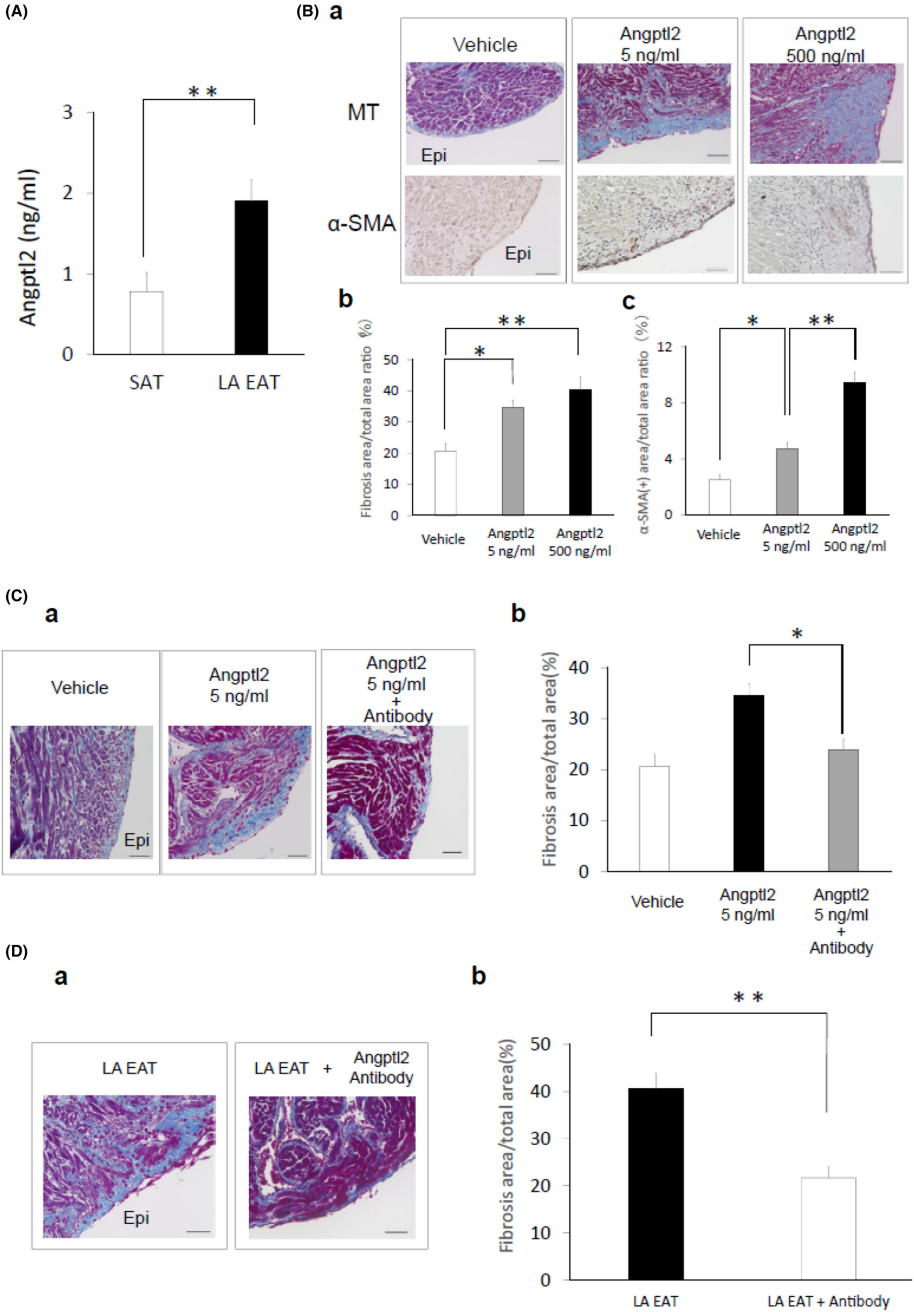
Effects of human recombinant Angptl2 and anti‐Angptl2 antibody. (A) Angptl2 protein expression with exposure to the human EAT and SAT‐conditioned media. The data are presented as the mean ± SEM (*n* = 9 for each group). *p < .05. (B) (a) Representative results for Masson's trichrome staining and immunohistochemical staining using α‐SMA antibody. Scale bar = 50 μm. (b) Quantification of epicardial fibrosis (*n* = 5 for each group). The data are presented as the mean ± standard error of the mean. *p < .05, **p < .01. (c) Quantification of α‐SMA‐positive cells (*n* = 5 for each group). The data are presented as the mean ± SEM. *p < .05, **p < .01. (C) (a) Representative images of left atrial sections from rats stained with Masson's trichrome. Scale bar = 50 μm. (b) Quantification of epicardial fibrosis (*n* = 5 for each group). The data are presented as the mean ± SEM. *p < .05. (D) (a) Representative images of left atrial sections from rats incubated with the EAT‐conditioned medium with or without anti‐Angptl2 antibody and stained with Masson's trichrome. Scale bar = 50 μm. (b) Quantification of epicardial fibrosis (*n* = 9 for each group). The data are presented as the mean ± SEM. **p < .01. EAT, epicardial adipose tissue; SAT, subcutaneous adipose tissue. (Figure adapted with permission^11^).

As shown in Figure 8A, Angptl2 protein expression in the peri‐LA EAT‐conditioned medium was higher than in the SAT‐conditioned medium. The effects of treatment of organo‐cultured rat atrium with human recombinant Angptl2 for 7 days are shown in Figure 8B. Figure 8B(a) depicts the representative results of Masson's trichrome staining and immunohistochemical staining using α‐SMA antibody. Human recombinant Angptl2 applied from the epicardial side induced fibrosis. The fibrotic area of the atrium treated with Angptl2 at doses of 5 and 500 ng/ml was greater than that treated with vehicle (Figure 8B(b)). Furthermore, the area of α‐SMA‐positive cells treated with Angptl2 at doses of 5 ng/ml was greater than that treated with vehicle. The area treated with 500 ng/ml was greater than the area treated with 5 ng/ml or vehicle (Figure 8B(c)). Treatment with human recombinant Angptl2 at a dose of 5 ng/ml in combination with anti‐Angptl2 antibody at a dose of 50 ng/ml reduced the fibrotic area of the organo‐cultured atrium compared with Angptl2 treatment alone (Figure 8C(a),(b)). Treatment with human EAT in combination with anti‐Angptl2 antibody at a dose of 5000 ng/ml reduced the fibrotic area of the organo‐cultured atrium compared with EAT treatment alone (Figure 8D(a),(b)).

A previous study showed that Angptl2 is associated with obesity‐related systemic insulin resistance and inflammation.^25^ As shown in Figure 8, application of human recombinant Angptl2 instead of EAT clearly induced atrial fibrosis in organo‐cultured rat atria. It is of note that the dose of human recombinant Angptl2 (5 ng/ml) was not far from the concentration of Angptl2 in the EAT (1.90 ng/ml) (Figure 8A). Together with the evidence showing that the fibrotic effects of both human recombinant Angptl2 (5 ng/ml) and human LA EAT were effectively suppressed by concomitant treatment with Angptl2 antibody, it was strongly suggested that the physiological protein concentration of Angptl2, as observed in EAT‐conditioned medium,^11^ is sufficient to induce proinflammatory atrial fibrosis in humans. Therefore, antagonizing the expression of Angptl2 in the EAT may be a novel therapeutic approach to prevent AF.

## 5 DETECTION OF FIBROTIC EAT REMODELING BY CT

As described previously,^10^ fibrotic EAT remodeling is associated with atrial myocardial fibrosis, proinflammatory cytokines/chemokines, and AF progression. In our previous study, we non‐invasively detected fibrotic EAT remodeling.^12^

### 5.1 Correlation of adipocyte diameter with body mass index (BMI) and EAT volume

**FIGURE 9:**
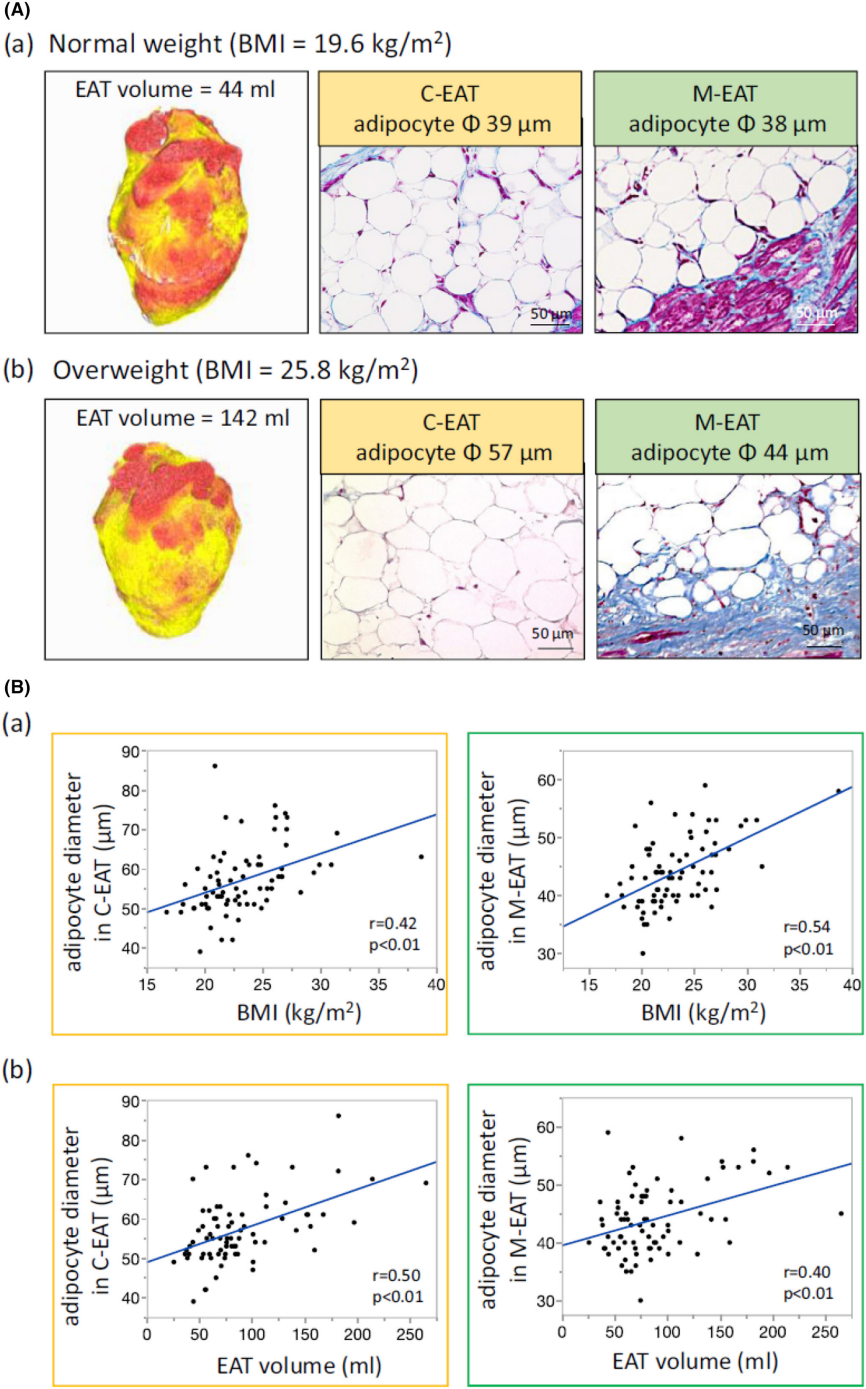
Relationship of adipocyte diameter with BMI and EAT volume. (A) (a,b) Representative examples. (B) (a,b) Correlation of adipocyte diameter with BMI and EAT volume. BMI, body mass index; EAT, epicardial adipose tissue. (Figure adapted with permission^12^).

We divided the EAT into the central EAT (C‐EAT) and the marginal EAT (M‐EAT). The C‐EAT was defined as the central area of the EAT that was not attached to either the myocardium or the epicardium, while the M‐EAT was defined as the area within 150 mm from the myocardium, including the adipocytes in contact with the myocardium. CT images and Masson's trichrome staining of samples from two representative patients (normal weight and overweight) are shown in Figure 9A. The EAT volume in the patient with a normal weight was 44 ml. The diameter of the C‐EAT (39 μm) was comparable to that of the M‐EAT (38 μm) (Figure 9A(a)). In contrast, in the patient who was classified as overweight, the EAT volume was 142 ml. The diameter of the C‐EAT (57 μm) was much greater than that of the M‐EAT (44 μm) (Figure 9A(b)). As shown in Figure 9B, in all 76 cases, the adipocyte diameter in both the C‐EAT and M‐EAT was positively correlated with BMI. Similarly, the adipocyte diameter in both the C‐EAT and M‐EAT was positively correlated with the EAT volume. Therefore, we emphasize the strong positive correlation between EAT fibrosis and the C/M adipocyte diameter ratio, which suggests that both larger adipocytes in the C‐EAT and smaller adipocytes in the M‐EAT were closely associated with the severity of fibrotic EAT remodeling.

### 5.2 Gene expression profile of the C‐EAT and M‐EAT by microarray analysis

**FIGURE 10:**
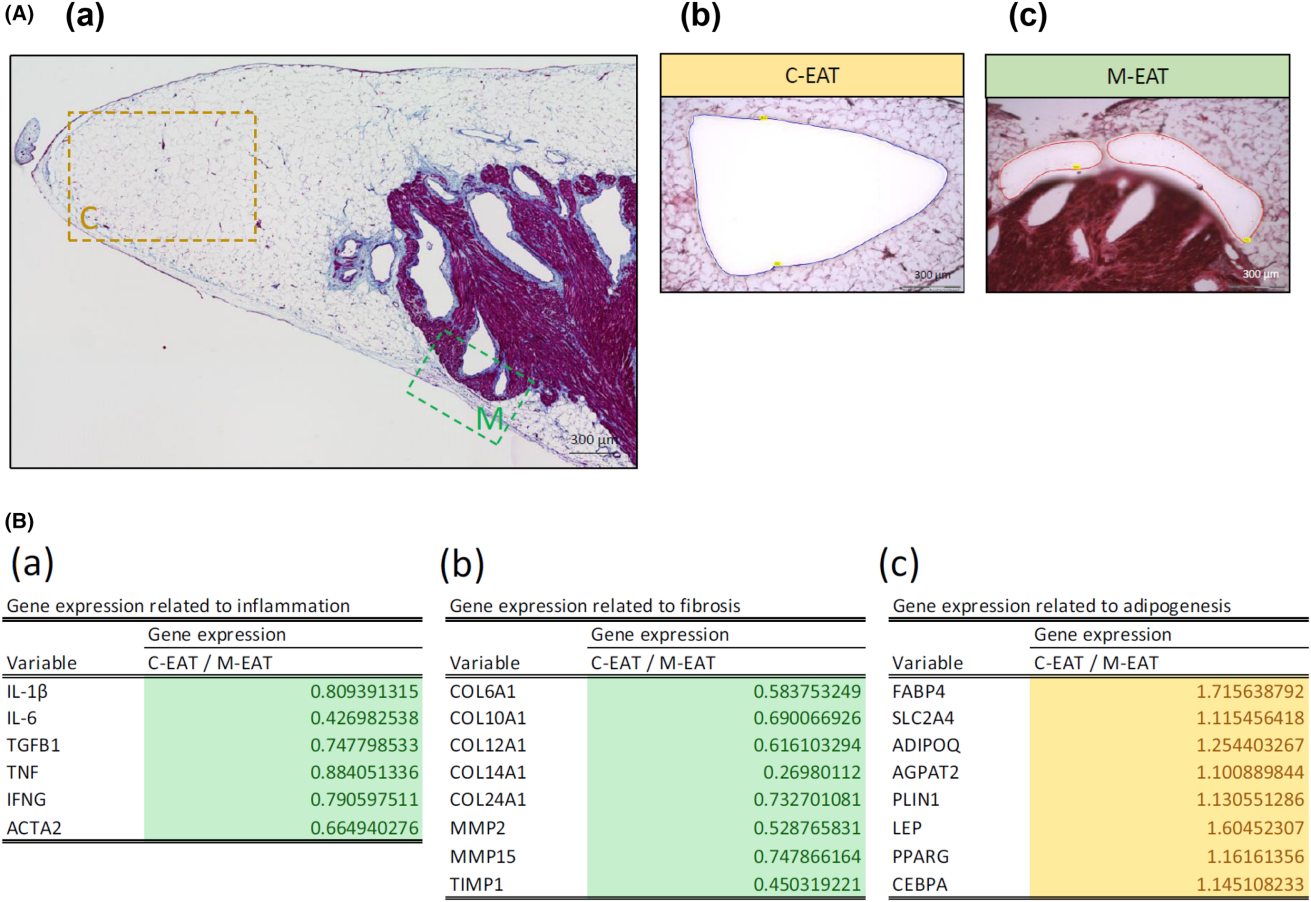
Micro‐dissection of the C‐EAT and M‐EAT for microarray analysis. (A) Laser micro‐dissection was performed on each adipose tissue in sections (C‐EAT: blue, M‐EAT: red), and used for the microarray analysis. (B) (a–c) Downregulation (green) and upregulation (yellow) of inflammation‐, fibrosis‐, and adipogenesis‐related genes in the C‐EAT compared with the M‐EAT. C, central; M, marginal (Figure adapted with permission^12^).

To test the hypothesis that specific cytokines in the M‐EAT may inhibit the normal development of pre‐adipocytes, resulting in smaller adipocytes in the M‐EAT, a microarray analysis of two distinct adipocyte clusters obtained from the C‐EAT and the M‐EAT was performed using adipocytes from three randomly selected patients. The EAT areas that were isolated and evaluated are illustrated in Figure 10A. The results of the microarray analysis are shown in Figure 10B(a–c). In these three cases, 871 genes were upregulated and 1741 genes were downregulated in the C‐EAT compared with the M‐EAT. Genes associated with inflammation, including the genes encoding IL‐6, transforming growth factor (TGF)‐β, and TNF, were downregulated in the C‐EAT (Figure 10B(a)). Genes associated with fibrosis, including COL, MMP, and TIMP, were also downregulated in the C‐EAT (Figure 10B(b)). Genes associated with adipogenesis, including *FABP4*, *PPARG*, and *CEBPA*, were upregulated in the C‐EAT (Figure 10B(c)).

### 5.3 Effects of proinflammatory cytokines on the expression of adipogenic and profibrotic genes in cultured human EAT

The EAT was isolated from the LAA of six randomly selected patients and cultured with the proinflammatory cytokines that were shown to be downregulated in the C‐EAT and upregulated in the M‐EAT (Figure 10B). The expression of mRNAs associated with adipogenesis after 24 h of exposure to these cytokines is shown in Figure 10B.

In agreement with Divoux et al,^36^ a recent review article^30^ concluded that visceral fibrosis may limit adipocyte expansion, thus acting as an adaptive mechanism to reduce the negative effect of adipocyte hypertrophy. This mechanism could explain the reduced adipocyte size in the M‐EAT. Lee et al^37^ recently demonstrated that glucocorticoids restrain cell‐autonomous TGF‐β signaling in adipose stem cells, thus facilitating adipogenesis and healthy remodeling in the abdominal SAT. These processes are impaired in omental adipose tissue. In the case of adipogenesis, we previously showed that the concentrations of IL‐6 and TNF‐α in the EAT were associated with atrial myocardial fibrosis.^10^ It is noteworthy that IL‐6 and TNF‐α inhibited normal pre‐adipocyte development and promoted the proinflammatory phenotype.^38^ A previous study showed that TGF‐β1 also reportedly caused differentiated adipocytes to revert to a state that is characteristic of pre‐adipocytes.^39^ In our microarray analysis, mRNAs corresponding to IL‐6, TNF‐α, and TGF‐β1 were more highly expressed in the M‐EAT than in the C‐EAT. Incubation of cultured EAT with IL‐6, TNF‐α, and TGF‐β1 also suppressed the expression of *FABP4*, *CEBPA*, and *PPARG* mRNA (Figure 10B(a–c)), all of which promote adipogenesis.^40^ Based on these findings, we suggested that IL‐6, TNF‐α, and TGF‐β1 contribute to the smaller size of adipocytes in the M‐EAT, at least in part, by adipogenesis suppression.

### 5.4 CT to determine the percent change in EAT fat attenuation

**FIGURE 11:**
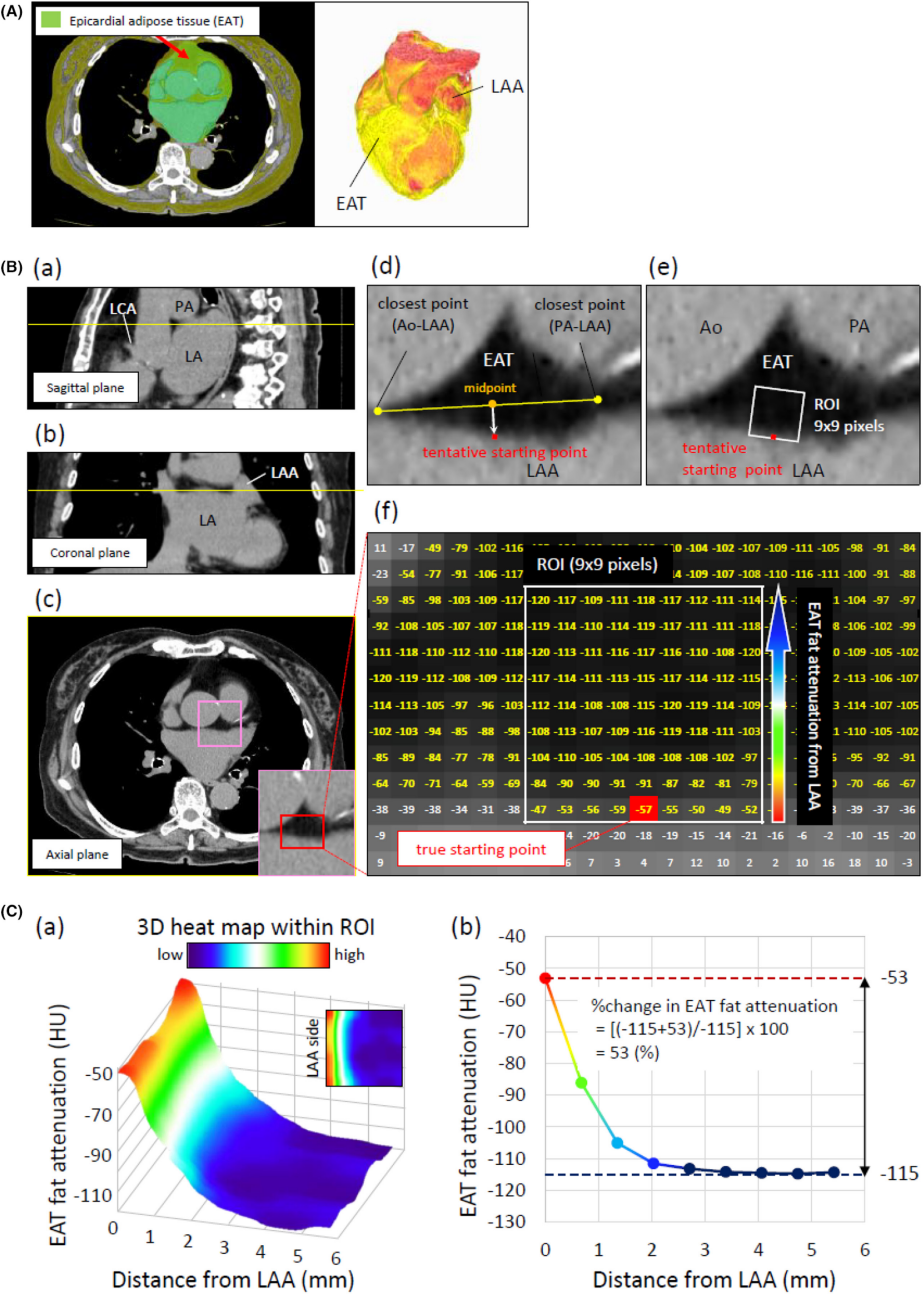
Quantification of EAT volume and percent change in EAT fat attenuation on CT. (A) Representative measurement image and three‐dimensional image of the EAT obtained with the Synapse Vincent system. (B) Representative measurement of EAT fat attenuation on CT. (a–c) CT images in the axial plane, including the maximum depiction of the EAT area above the origin of the left coronary artery and below the pulmonary artery bifurcation. (d–f) ROI determination. The starting point was determined as the point of intersection of the EAT edge close to the LAA and the perpendicular line from the midpoint between each closest point of the LAA to the aorta and the LAA to the pulmonary artery. As the ROI, 9 × 9 pixels were drawn from the edge line centered on the starting point to the center of the EAT. The yellow number is the EAT fat attenuation satisfying the window of −195 to −45 HU. (C) (a) Representative three‐dimensional heatmap of EAT fat attenuation within the ROI. (b) The curve derived from the EAT fat attenuation from the LAA toward the center of the EAT. The percent change in EAT fat attenuation was calculated as follows: percent change in EAT fat attenuation = 100 × (maximum CT fat attenuation−minimum CT fat attenuation) ÷ maximum CT fat attenuation. CT, computed tomography; LAA, left atrial appendage; LCA, left coronary artery; LA, left atrium; Ao, aorta; PA, pulmonary artery; ROI, region of interest. (Figure adapted with permission^12^).

To evaluate EAT fat attenuation using CT, a high‐speed three‐dimensional image analysis system (Synapse Vincent; Fuji Photo Film) was used. Two cardiologists who were blinded to all of the patient information reviewed the CT images. The EAT analysis was performed on axial images above the origin of the left coronary artery and below the pulmonary artery bifurcation where the cross‐sectional EAT area was at its maximum (dotted yellow line in Figure 11B(a–c)). First, the EAT was defined within a window of −195 to −45 HU. Second, the two closest LAA points to both the aorta and the pulmonary artery were determined, and a line was drawn between them (Figure 11B(d)). Another line was drawn perpendicular to the LAA from the midpoint of the abovementioned line. Then, a tentative starting point was set at the EAT edge close to the LAA. From the tentative starting point, bilateral lines parallel to the surface of the LAA were drawn. The 9 × 9 pixels were created from this line to the distal EAT (Figure 11B(e)). The mean CT fat attenuation for each pixel was calculated automatically (Figure 11B(f)). The 9 × 9 pixels were defined as the region of interest (ROI) because they were at a maximum range without interference from other structures, such as the aorta and pulmonary artery, in all subjects. Finally, the true starting point was adjusted to the most proximal nine pixels that showed a value of ≤−45 HU. The three‐dimensional heatmap was created for each patient within the 9 × 9 pixels (ROI) using ImageJ software (National Institutes of Health) (Figure 11C(a)). The curve was constructed to follow the mean EAT fat attenuation of each pixel line from the LAA toward the center of the EAT within the ROI (Figure 11C(b)). Following the previous study, the percent change in EAT fat attenuation from the maximum CT fat attenuation (proximal side from the LAA in the EAT) to the minimum CT fat attenuation (distal side) was calculated as follows: percent change in EAT fat attenuation = 100 × (maximum CT fat attenuation−minimum CT fat attenuation) ÷ maximum CT fat attenuation (Figure 11C(b)).

### 5.5 Percent change in EAT fat attenuation determined by CT and fibrotic EAT remodeling

**FIGURE 12:**
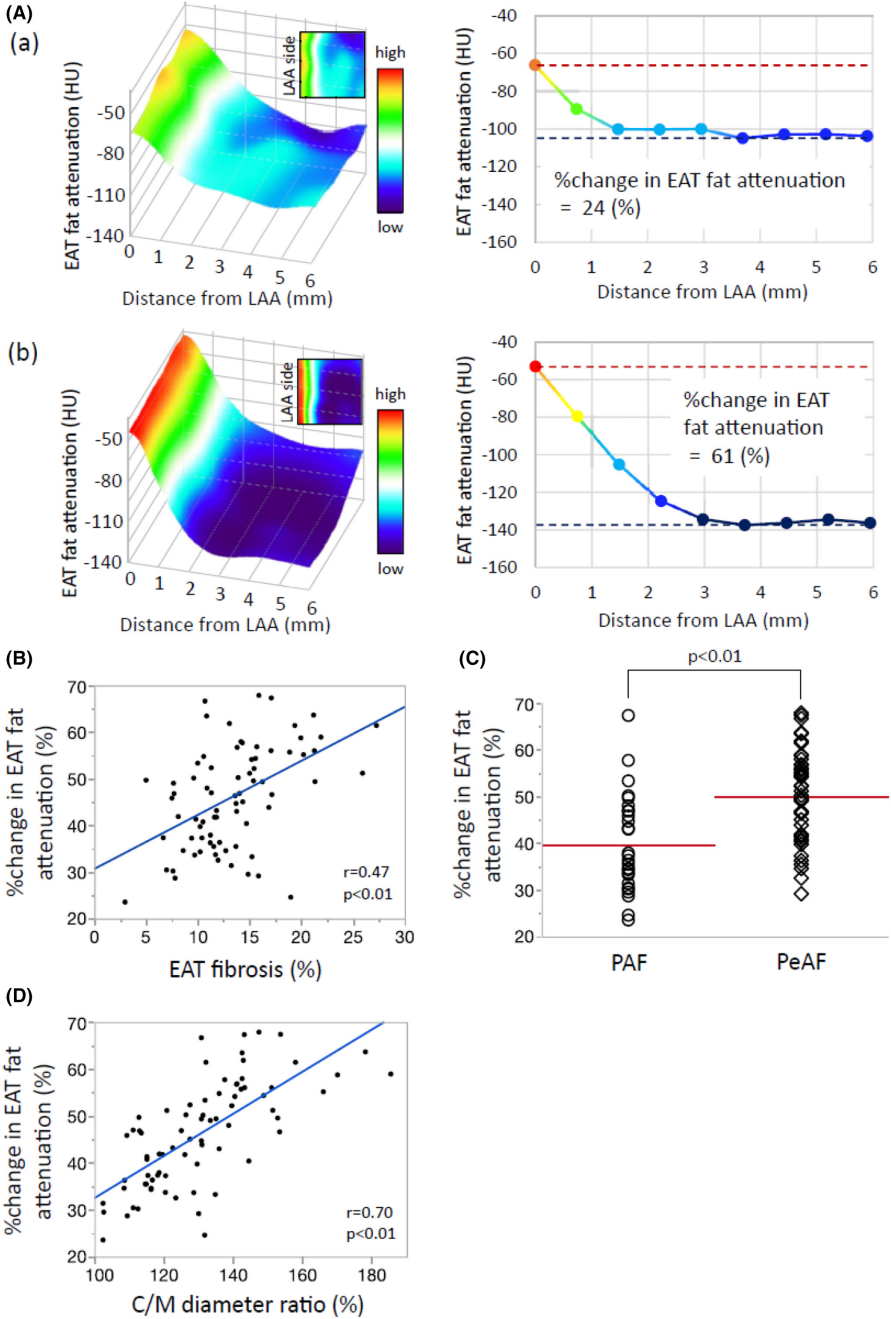
Percent change in EAT fat attenuation on CT imaging. (A) Representative heatmap of EAT fat attenuation in cases of mild (a) and severe (b) fibrotic EAT remodeling. (B) Correlation between the percent change in EAT fat attenuation and EAT fibrosis. (C) Comparison of the percent change in EAT fat attenuation between PAF and PeAF. (D) Correlation between the percent change in EAT fat attenuation and C/M diameter ratio. PAF, paroxysmal atrial fibrillation; PeAF, permanent AF. (Figure adapted with permission^12^).

Representative EAT fat attenuation on CT images is shown in Figure 12. In a patient with mild fibrotic EAT remodeling (EAT fibrosis 7.6%), the percent change in EAT fat attenuation was gradual and was calculated to be 24% (Figure 12A). In contrast, in a patient with severely fibrotic EAT remodeling (EAT fibrosis 27.3%), the percent change in EAT fat attenuation was large and was calculated to be 61% (Figure 12B). The percent change in EAT fat attenuation in all 76 patients was positively correlated with EAT fibrosis. The percent change in EAT fat attenuation was greater in the PeAF group than in the PAF group (Figure 12C) and was positively correlated with the histologically assessed C/M diameter ratio (Figure 12D). Based on these findings, we concluded that the central‐to‐marginal adipocyte diameter ratio was strongly associated with fibrotic EAT remodeling. In addition, the percent change in EAT fat attenuation determined by CT non‐invasively detected remodeling.^12^

CT images of adipose tissue with larger adipocytes showed lower attenuation because of the increased oil droplet size,^41^ while poorly differentiated smaller adipocytes showed higher attenuation on CT images.^42^, ^43^ Tissue inflammation and subsequent fibrosis can also increase tissue attenuation on CT.^44^ In a large cohort of patients undergoing cardiac surgery, Antonopoulos et al^45^ demonstrated that the average attenuation of adipose tissue was inversely correlated with adipogenic gene expression and average adipocyte size, which is driven by intracellular lipid accumulation. More recently, Goeller et al^46^ showed that the attenuation of peri‐coronary adipose tissue measured by routine CT angiography was related to the progression of non‐calcified plaques. This concept can also be applied to a study by Ciuffo et al,^47^ who used the mean CT attenuation in the standard four‐chamber view to measure the quality and quantity of peri‐LA fat tissue by area (mm^2^) in 143 consecutive patients with drug‐refractory AF who were referred for their first catheter ablation for AF. Because LA fat attenuation correlated with the peri‐LA fat volume and was associated with AF recurrence, the authors concluded that peri‐LA fat attenuation assessments can improve AF ablation outcomes by refining patient selection.^47^ Our study may be more valuable because it compared the EAT fat attenuation on CT imaging with histological findings in each patient.^12^ In fact, the percent change in EAT fat attenuation on CT was well correlated with the histologically assessed C/M diameter ratio (Figure 12D). The results of our study suggested that fibrotic EAT remodeling promoted atrial myocardial fibrosis and AF, which could be detected by CT imaging. Many patients undergo coronary artery CT angiography examinations, and the evaluation of EAT fat attenuation in these individuals may identify subjects who are at a high risk of new AF development.

## 6 CONCLUDING REMARKS

In the latter part of this special review article, we summarized the main results of three of our previous studies. Together with the results of the systematic review, it is obvious that proinflammatory EAT is associated with AF recurrence after catheter ablation in patients with AF. From the clinical viewpoint, it is very important to prevent EAT accumulation to reduce new‐onset AF, as well as to reduce AF recurrence after catheter ablation. Cardiac CT is performed in all patients who undergo catheter ablation; thus, the quantity and quality of EAT can be evaluated using CT in these patients. CT can also be used for risk stratification to predict AF recurrence before catheter ablation.

## CONFLICT OF INTEREST STATEMENT

Authors declare no conflict of interests for this article.
